# Evaluating the pharmacological activities of *Aloe perryi*–Silver nanoparticles induced apoptosis against colon cancer cells (HCT‐116)

**DOI:** 10.1002/fsn3.4246

**Published:** 2024-05-27

**Authors:** Omar Hotan, Ali Alhaj, Abdulghfor Al‐quhaim, Khaled Alburaihi, Yahya Ahmed, Qassem Munasser, Saleh Bin Dhufer, Tammam Nasran, Mohammed Gabir, Akram Ebrahim, Mohammed Obadi, Maryam Hadi, Hanefa Al‐baity, Abdulmalek Ba‐Nafea, Eskandar Qaed, Mohamed Y. Zaky, Mohammed Okba, Abdullah Al‐Nasi, Marwan Almoiliqy

**Affiliations:** ^1^ Department of Pharmacy, Faculty of Medicine and Health Sciences University of Science and Technology Aden Yemen; ^2^ Microbiology Department Supreme Commission for Drug and Medical Appliances Aden Yemen; ^3^ State Key Laboratory of Applied Organic Chemistry College of Chemistry and Chemical Engineering, Lanzhou University Lanzhou Gansu China; ^4^ Molecular Physiology Division, Zoology Department, Faculty of Science Beni‐Suef University Beni‐Suef Egypt

**Keywords:** *Aloe perryi*, apoptosis, characterization, pharmacological activities, silver nanoparticles, synthesis

## Abstract

*Aloe perryi* has been studied and possesses several activities, including antibacterial, antiparasitic, and anticancer properties. In this study, *A. perryi* was used as a reducing agent of silver ions into silver nanoparticles. *Aloe perryi–*silver nanoparticles (APS‐NPs) were characterized and evaluated using characterization techniques. However, the antioxidative, antibacterial, and anticancer assays were studied to evaluate the pharmacological activities of APS‐NPs. APS‐NPs were developed and changed to dark brown and the maximum absorption was 442 nm. SEM (5–583 nm), TEM (4–110 nm), XRD (21.84 nm), and zeta potential analysis (63.39 nm) revealed that the APS‐NPs were nano‐sized, and the APS‐NPs had a cubic crystalline structure, according to the XRD results. FTIR analysis suggested that functional groups of *A. perryi* metabolites were involved in forming APS‐NPs. The zeta potential indicated that the APS‐NPs were negatively charged (−32 mV), suggesting good stability. APS‐NPs showed significant antioxidative stress activity by reducing DPPH‐free radicles in a dose‐dependent manner. APS‐NPs‐induced antibacterial effect against *Staphylococcus aureus* (*S. aureus*), *Escherichia coli* (*E. coli*), and *Acinetobacter baumannii* (*A. baumannii*). APS‐NPs reduced the cell viability and cell migration of the human colon tumor cell line (HCT 116) compared with controls, indicating that APS‐NPs could play a role in reducing metastasis and inducing cell apoptosis against colon cancer. In conclusion, the nanoparticle synthesis from *A. perryi* extract demonstrated excellent antioxidant, antibacterial, and anticancer activities, thus suggesting that our APS‐NPs have the potential to be used as antioxidative and antibacterial in food and pharmaceutical industries.

## Graphical abstract

Synthesis of silver nanoparticles via using *Aloe perryi* as a reducing agent, and illustrating the characterization studies of *Aloe perryi–*silver nanoparticles and their pharmacological activity against HCT‐116 colon cancer cells.

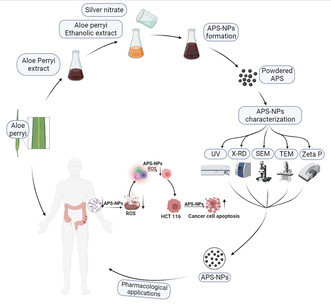

## 1 INTRODUCTION

The exponential development of nanotechnology has led to a rise in the use of numerous products using nanoparticles in various fields, including food science, cosmetics, and medicine (Mohammad et al., 2022). The particle size range of biotechnology‐related nanoparticles is 10–500 nm, seldom going over 700 nm (Mody et al., 2009). The nanoscale size of these particles allows them to communicate with biological molecules on cell membranes and inside cells in a way that can be decoded and attributed to these cell's biochemical and physical attributes. It also allows them to penetrate deeper into the cells (Mody et al., 2009). It also extensively uses nanoparticles in several fields, including electronics, cosmetics, diagnostic, and therapeutic applications (Missaoui et al., 2018).

The capacity to detect nanomaterials using methods that have atomic resolution abilities, like transmission electron microscopy, tandem electron microscopy, and scanning electron microscopy, has contributed to the tremendous expansion and interest that have been seen in nanotechnology (Sharma et al., 2019). Nanoparticles potentially act as drug carriers for usage in diagnostic or therapeutic applications within the pharmaceutical industry. It is advised that these nanoparticles, which include nanoemulsions, liposomes, polymeric nanoparticles, and solid nanoparticles, have potential therapeutic uses relevant to their chemical and physical characteristics, drug release, drug loading efficiency, and toxicity of the carrier itself (Akbari et al., 2022). Unfortunately, many medications with therapeutic effectiveness have low solubility in aqueous environments. Encapsulating these pharmaceuticals in nanoparticles, on the other hand, may increase their stability by reducing the likelihood of precipitation and the amount of toxic cosolvents required.

Additionally, nanoparticles have the ability to alter the rate of medication metabolism and clearance, which leads to an increase in medicinal effectiveness (Bao et al., 2015). The most prevalent form of nanoparticles is made of metal (metal nanoparticles) and has found widespread use in various industries, including agriculture, food, cosmetics, and medicine (Seçkin, 2021; Seckin et al., 2022). One of the most common approaches to synthesizing metal nanoparticles is a chemical reduction of the noble metals. This is one of the various ways metal nanoparticles can be produced (Habibullah et al., 2021).

Silver nanoparticles are increasingly being characterized by a wide variety of applications, including thermal, electrical, optical, high electrical conductivity, and biological applications. These applications are directly related to the unique chemical, physical, and biological properties that silver nanoparticles possess (Xu et al., 2020). The primary basis for treating bacterial infections is antibiotics. However, the drawbacks of antibiotic use, including antibiotic resistance, antibiotic‐associated adverse effects, immune suppression, allergic responses, and a decrease in beneficial bowel and mucosal microbiota, have triggered a strong interest among scientists in creating less toxic, more potent, and non‐anti‐infective antibiotics (Ramirez et al., 2020). Silver ion has a wide range of antibacterial actions owing to its high inhibitory and bactericidal effects (Woo et al., 2008).

The fabricated silver nanoparticles offer remarkable antibacterial properties, particularly in comparison with an antibiotic (Alqahtani et al., 2020). Silver nanoparticles' antimicrobial properties have been demonstrated to have broad‐spectrum effects against gram‐negative and gram‐positive bacteria, fungi, and viruses (Dakal et al., 2016; Lee et al., 2016). However, the drawbacks of silver nanoparticles synthesized chemically could be due to reducing agents' toxicity, expense, low reducing ability, and ease of introducing additional contaminants to the process (Gudikandula & Maringanti, 2016).

The biogenic (green chemistry) technology of metal nanoparticle production, which uses natural resources including plants and microorganisms, has been offered as a viable adjunct to conventional methods of synthesis (Ahmed et al., 2016, 2022; Singh et al., 2018). Fungi and bacteria are recognized as the types of microorganisms responsible for creating a crucial role in the eradication of hazardous chemicals by inducing reduction of metal ions (Bahrulolum et al., 2021; Lahiri et al., 2021). It has been demonstrated that several distinct types of bacteria are capable of producing silver nanoparticles within their cells, where the components of the cell may act as both stabilizing and reducing agents (Al‐Oqail et al., 2016).

However, the process of stabilizing the silver nanoparticles uses natural capping agents rather than synthetic ones (Javed et al., 2020). For instance, silver nanoparticles were synthesized using gram‐positive bacteria, fungi, and plant‐synthesized silver nanoparticles, which have become of high interest to scientists (Gur, 2022). Using the agar well diffusion assay, *Lactobacillus bulgaricus* showed antibacterial activity against selected pathogenic strains, that is, *Staphylococcus epidermidis* (*S*. *epidermidis*), *Salmonella typhi* (*S*. *typhi*), and *Staphylococcus aureus* (Naseer et al., 2022). The green‐synthesized silver nanoparticles using *Saccharomyces boulardii* yeast provided nanoparticles with anticancer activity (Kaler et al., 2013). In vitro, silver nanoparticles synthesized by *Aspergillus sydowii* demonstrated significant antifungal activity against a variety of clinical pathogenic fungi and apoptotic activity in HeLa and MCF‐7 cells (Wang et al., 2021).

Socotrine *Aloe*, also known as *Aloe perryi* (*A*. *perryi*), is a species of plant in the genus *Aloe* (*Asphodelaceae*). *Aloe perryi* is grown in the Arabian peninsula, and it is endemic to the Socotra island in Yemen. Because of its name, it is often known as Socotrine *Aloe* (Al‐Gabri et al., 2022). Traditional medicine has extensively studied *A. perryi* extracts, which have several applications, including antibacterial, antiparasite, and anticancer application (Al‐Gabri et al., 2022). Kumar et al. (2019) discussed that the *A. perryi* and *Aloe vera* (*A*. *vera*) methanolic extracts were rich in phytochemicals, including anthraquinones, anthrones, flavonoids, and chromones, although the amounts of all these phytochemicals were higher in *A*. *perryi*. However, *A. perryi* and *A. vera* methanolic extracts also have significant antioxidative stress and antidiabetic effects. The unique compound of the *Aloe* species, which has not been found in any other plant, is C‐glycosylated chromone, a phenolic compound that possesses antioxidative stress, antimicrobial, anticancer, and anti‐inflammatory activities (Badoni et al., 2020). Several studies have found that *A. perryi* has various therapeutic effects, such as antioxidative stress, antidiabetic, anti‐inflammatory, antibacterial, and antitumor effects (Al‐Fatimi et al., 2007; Suliman et al., 2021). In the current study, we proposed that *A. perryi* was primarily responsible for reducing silver ions and forming silver nanoparticles. We speculated studying the pharmacological activity of prepared silver nanoparticles by using *A*. *perryi*, including antibacterial and antioxidative stress, and evaluating their cytotoxic effect against colon cancer cells (HCT‐116).

## 2 MATERIALS AND METHODS

### 2.1 Materials

*Aloe perryi* extract was purchased from Socotra Island local market, Yemen; HCT‐116 cell line was purchased from the American Type Culture Collection (ATCC), USA; *Escherichia coli*, *S. aureus*, and *Acinetobacter baumannii* were isolated from anonymous samples of hospitalized patient in Almadeinah Medical Center, Aden, Yemen; PRMI 1600 medium, fetal bovine serum (FBS), and bovine insulin were obtained from Biowest south American, USA; Silver nitrate was obtained from Emsurr, USA; DPPH kit was obtained from Sigma, USA; Mueller–Hinton agar was purchased from Himedia, India; DMSO was obtained from Analar, Ireland; Ciprofloxacin, Erythromycin, and Vancomycin were purchased from Himedia, India; and penicillin/streptomycin were obtained from Biowest south American, USA. Other chemicals were used as an analytical grade.

### 2.2 Methods

#### 2.2.1 Plant collection and extraction

The Socotrian *Aloe*, or *A. perryi* extract, has been purchased from the Socotra Island local market in Yemen. Here, we briefly described the traditional method of *A. perryi* leaf extraction. The whole leaf was collected from the base of the plant. Then, the leaf was cut and dropped into a traditional animal pan for several days. After that, the extract was allowed to dry for up to 2 months before being stored in a dry location for further usage (Aloe Unique Tapping Process, 2015; AloeF143, 2018; Alotaibi, 2022).

#### 2.2.2 Synthesis of APS‐NPs by using *A. perryi*

Various methods for the production of nano‐scaled materials are accessible in the natural environment (Mohanpuria et al., 2008). Several factors influence the choice, including the concept of synthesizing nanoparticles from metal salts using a green approach rather than a conventional method (Patra & Baek, 2014). In this research, our nanoparticles were prepared using *A. perryi* liquid extract and 5 mM silver nitrate solution (AgNO_3_), providing APS‐NPs following the previously prescribed method with a little fabrication (Ahmed et al., 2019). The alcoholic solution of ethanol and *A. perryi* extract was first prepared in a ratio of 95:5, respectively. The mixture of aqueous AgNO_3_ and alcoholic *A. perryi* solutions was also prepared in a ratio of 90:10, respectively. Then, the mixture was heated at 60°C at 200 rpm, stirring for up to 4 h till the color changed to dark brown. The color intensity was observed to increase until no more changes were noticed, which was interpreted as an indication of the entire biotransformation of silver ions into silver nanoparticles. The APS‐NPs dispersion was centrifuged at 4000 rpm/15 min to exclude the residual solution and get the precipitated nanoparticles. Then, the prepared APS‐NPs were washed with deionized H_2_O and centrifuged three times at 4000 rpm for 5 min. After centrifuging, the freshly prepared APS‐NPs were oven‐dried at 45°C and stored for further experiments.

#### 2.2.3 Characterization studies of APS‐NPs

The ultraviolet–visible spectrum of the synthesized APS‐NPs was checked in the range of 200–800 nm using an ultraviolet–visible–near‐infrared spectrophotometer (Cary Series UV–Vis–NIR, Australia) to measure the maximum absorbance (λ_max_) of synthesized APS‐NPs. The maximal absorbance was determined by diluting 1 mL of produced APS‐NPs with 2 mL of dd H_2_O. The crystallinity and lattice parameters of APS‐NPs were measured using an X‐ray diffractometer (Empyrean Malvern Panalytical, Netherlands) at 40 kV and 30 mA. The XRD crystal size was calculated by using Scherrer's Equation. In this study, APS‐NPs charge and distribution were measured by using the Zetasizer Nano‐ZS (vertern Model ZEN3600, d, Malvern, UK). FTIR experiment was used to identify the functional groups and molecules that are vital in the reduction of silver ions into silver nanoparticles (Levin & Bhargava, 2005). Our samples were scanned in spectra 4000–400 cm^−1^ and a resolution of 4 cm^−1^ by using FTIR (Vertex 70 Ram II, Germany). Several peaks were recorded and recognized to detect the functional groups present on the purified developed APS‐NPs. Scanning electron microscopy (SEM) characterization was performed in this study by an SEM (Quattros, Thermo Scientific, Netherland). Two images of APS‐NPs were taken, each with a different level of magnification (this can be seen by comparing the various markers that appear in each image). The precise shape and size of the produced APS‐NPs were identified by transmission electron microscopy (TEM), which was performed on a TEM apparatus (JEOL JEM‐2100, Japan) equipped with a high‐resolution objective pole piece and operated at acceleration voltages of 200 kV. The ImageJ program (National Institute of Mental Health, Bethesda, Maryland, USA) was used to calculate the average diameter, size distribution, particle count, and dimension of the nanoparticles based on the SEM and TEM images. OriginPro 2018 was used to carry out the statistical analysis in additional detail (OriginLab, Northampton, MA, USA).

#### 2.2.4 Quantitative antioxidative stress activity by DPPH radical

In this study, we performed the DPPH free radical scavenger activity to detect the antioxidative stress activity of our developed APS‐NPs as previously described (Melkamu & Bitew, 2021). Two milliliters of 0.1 M DPPH methanolic solution was mixed with 1 mL of various dilutions of APS‐NPs (50 μg, 100 μg, 200 μg, 400 μg, 800 μg, 1600 μg/mL), then allowed to incubate for 30 min at 25°C in the dark and detected at 517 nm using ultraviolet–visible–near‐infrared spectrophotometer (Cary Series UV–Vis–NIR, Australia). Methanol was used as a blank, and methanol + DPPH was used as a control in the experiment. The inhibition rate of DPPH free radicals by the samples was calculated according to the following equation (Balaji et al., 2011):

$$\text{DPPH scavenging activity}\;\left(\%\right)=\left[\frac{A_{c517}-A_{s517}}{A_{c517}}\right]\times 100$$

where *A*_c517_ is the absorbance of the control at 517 nm, and *A*_S517_ is the absorbance of sample at 517 nm.

#### 2.2.5 Antibacterial test

The APS‐NPs were investigated for their ability to inhibit bacterial growth using agar well diffusion. The Mueller–Hinton Agar medium was used for this assay. The 100 mL of molten Mueller–Hinton agar was homogenized and poured into the Petri dishes until it became dry. In this study, anonymous samples of pathogenic bacteria were isolated from hospitalized patients in Almadeinah Medical Center, Aden, Yemen. The bacteria (*E. coli*, *A. baumannii*, and S. *aurous*) were activated by inoculating a loop full of each strain in sterile distilled water. They were incubated at room temperature until they reached a required concentration of 10^8^ cells/mL per the McFarland standard. Then, the bacteria were inoculated in 100 mL of molten Mueller–Hinton agar media. After that, wells were made in the seeded plates with the help of sterile pipets, and each well was filled with 50 μL of various dilutions of APS‐NPs of 0.2, 4, 8, and 16 mg/mL dissolved in DMSO. Ciprofloxacin 5 μg, Erythromycin 15 μg, and Vancomycin 30 μg were the positive controls, and DMSO was the negative control. Microbial growth was measured by the diameter of the inhibitory zone (Parekh & Chanda, 2007).

#### 2.2.6 Cell culture

Human tumor cell line HCT‐116 was purchased from ATCC (American Type Culture Collection) and cultured according to the manufacturer's instructions. HCT‐116 cells were grown in RPMI 1640 medium with 10% FBS, 1% penicillin/streptomycin, and 0.1 U/mL bovine insulin. Afterward, HCT‐116 cells were allowed to incubate in a 5% CO_2_ incubator (NuAire, USA) at 37°C for 24 h before cytotoxicity and wound healing assays.

#### 2.2.7 Cell viability assay

The HCT‐116 cells were seeded at a density of 1 × 10^4^ cells per well in 100 μL of RPMI 1640 media in 96‐well plates and allowed to grow overnight. After cells were treated with APS‐NPs at different concentrations (6.25, 12.5, 50, 100, 200, 400, 800, 1600, and 2400 g/mL), they were allowed to incubate at 37°C with 5% CO_2_ for 24 h. As described previously, the MTT viability experiment was conducted with minor modifications (Mosmann, 1983). First, a 5 mg/mL stock solution of MTT in phosphate‐buffered saline (PBS, pH 7.2) was prepared and filtered. At the end of the treatment period (24 h), 20 μL of MTT solution was added to each well and allowed to incubate for 4 h at 37°C, and then 100 μL of DMSO was added to solubilize the crystals of formazan in each well while shaking. The absorbance measurements were then collected using a microplate reader at 570 nm (Biotek elx 800, USA). The live cells generated a dark blue formazan result, but the dead cells did not create any staining. The percentage of the viable cells was calculated using the following formula:

$$\text{Cell viability}\;\left(\%\right)=\left[100\times \left(\text{sample}\;\mathrm{abs}\right)/\left(\text{control}\;\mathrm{abs}\right)\right]$$

#### 2.2.8 Wound healing assay

The capacity of HCT‐116 to migrate was examined using a wound healing test. With RPMI 1640 culture media, HCT‐116 cell suspensions were cultured at a density of 5 × 10^4^ on 24‐well plates (Corning, USA) and incubated until the cell confluence reached 90%. Using a pipette tip of 200 μL, a straight line was drawn on the well's surface to create a scratch model. Exfoliated cells were then washed away with PBS. Adherent cells were exposed to APS‐NPs at 370 μg /mL for 0, 24, and 48 h, after which the scratch width was manually examined and quantified.

#### 2.2.9 Statistical analysis

For data charting and analysis, GraphPad Prism 8.0.2 (GraphPad Prism Software, La Jolla, CA) and OriginPro 2018 software (OriginLab, Northampton, MA, USA) were used. Malvern HighScore software was used to do the XRD analysis (Malvern PANalytical X‐ray Company, Almelo, Netherland). Additionally, SEM and TEM examinations were conducted using ImageJ software (National Institute of Mental Health in Bethesda, Maryland, USA). The analysis of variance (ANOVA) across groups was conducted using one‐way and two‐way ANOVA. In three separate experiments, all biological data were collected, and they were all reported as the mean standard deviation (SD). The statistical significance of the differences was indicated by *p*‐value of .05.

## 3 RESULTS

### 3.1 Characterization of APS‐NPs by using ultraviolet–visible spectrophotometer

**FIGURE 1:**
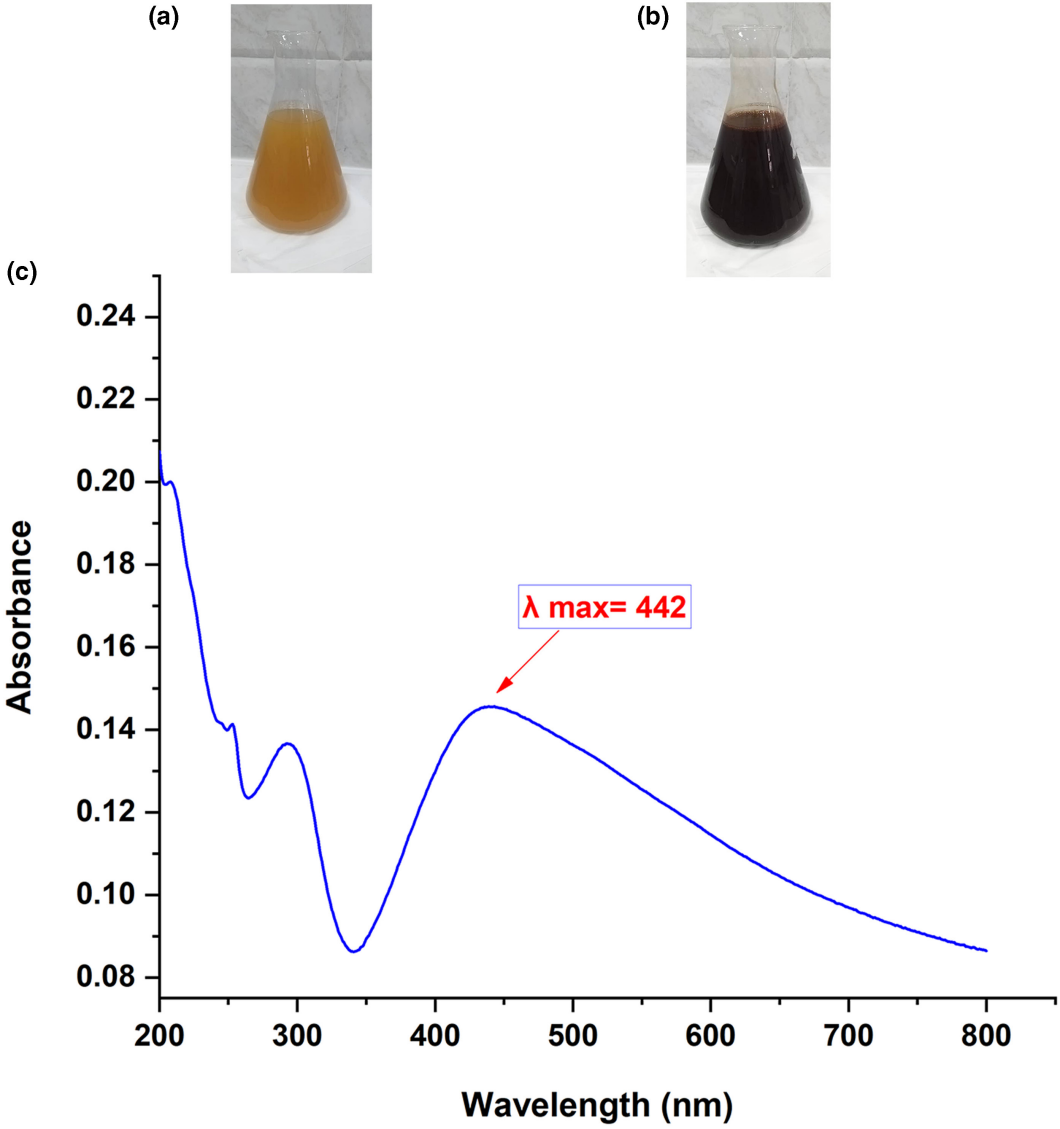
Characterization of APS‐NPs via detection of the color change and maximum absorption: (a) the mixture of ethanolic extract of *Aloe perryi* extract and AgNO_3_ at 0 time of the reaction. (b) The dispersion of ethanolic extract of *A. perryi* extract and AgNO_3_ at 4 h of the reaction. (c) The spectrum of maximum absorbance of developed APS‐NPs. APS‐NPs, *Aloe perryi‐*silver nanoparticles.

The typical indicator of the green formation of silver nanoparticles with plant extract is a transition in color to dark brown (Melkamu & Bitew, 2021). Our findings indicated that after 4 h of the reaction, the color of the *A. perryi* extract and AgNO_3_ mixture had changed from light orange to dark brown, indicating the effective synthesis of APS‐NPs (Figure 1a,b). The maximum absorbance of green‐developed silver nanoparticles was found in many investigations to be between 400 and 500 nm, confirming the silver nanoparticle synthesis (Almalki & Khalifa, 2020; Al‐Sanea et al., 2021). Our results showed that the maximum absorption of APS‐NPs was at 442 nm, similar to the range in the literature, indicating the formation of APS‐NPs successfully (Figure 1c), suggesting that *A. perryi* phytochemicals could play the main role in targeting the reduction of silver ions into silver nanoparticles.

### 3.2 Characterization of APS‐NPs morphology by using SEM

**FIGURE 2:**
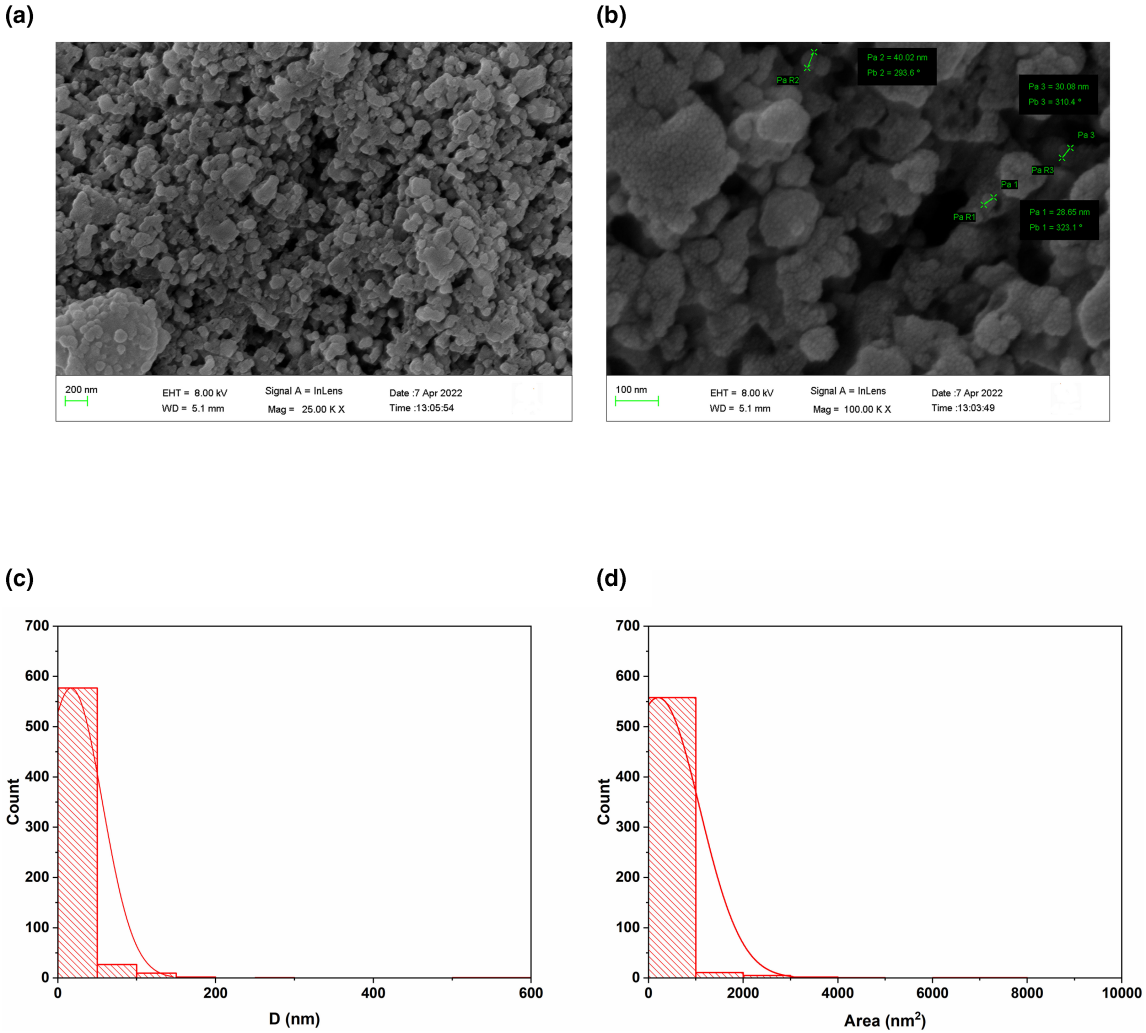
Characterization of APS‐NPs by using SEM technology: (a, b) SEM images of APS‐NPs synthesized using *Aloe perryi* extract showing the particle size range from 5 to 583 nm. (c) Histogram of particle size distribution (diameter) of APS‐NPs analyzed by ImageJ software, (d) Histogram of area measurement of APS‐NPs analyzed by ImageJ software. APS‐NPs, *Aloe perryi‐*silver nanoparticles.

SEM analysis was used to analyze the APS‐NPs for screening their structure and morphology. Our findings showed that APS‐NPs were nano‐sized (Figure 2a,b), with a minimum size of 5 nm and a maximum size of 583 nm. Furthermore, SEM histograms of particle size distribution and area of APS‐NPs revealed that the average size and area of APS‐NPs were nearly 16.8 and 200 nm^2^, respectively, indicating that APS‐NPs were significantly nano‐sized (Figure 2c,d).

### 3.3 Characterization of APS‐NPs morphology by using TEM

**FIGURE 3:**
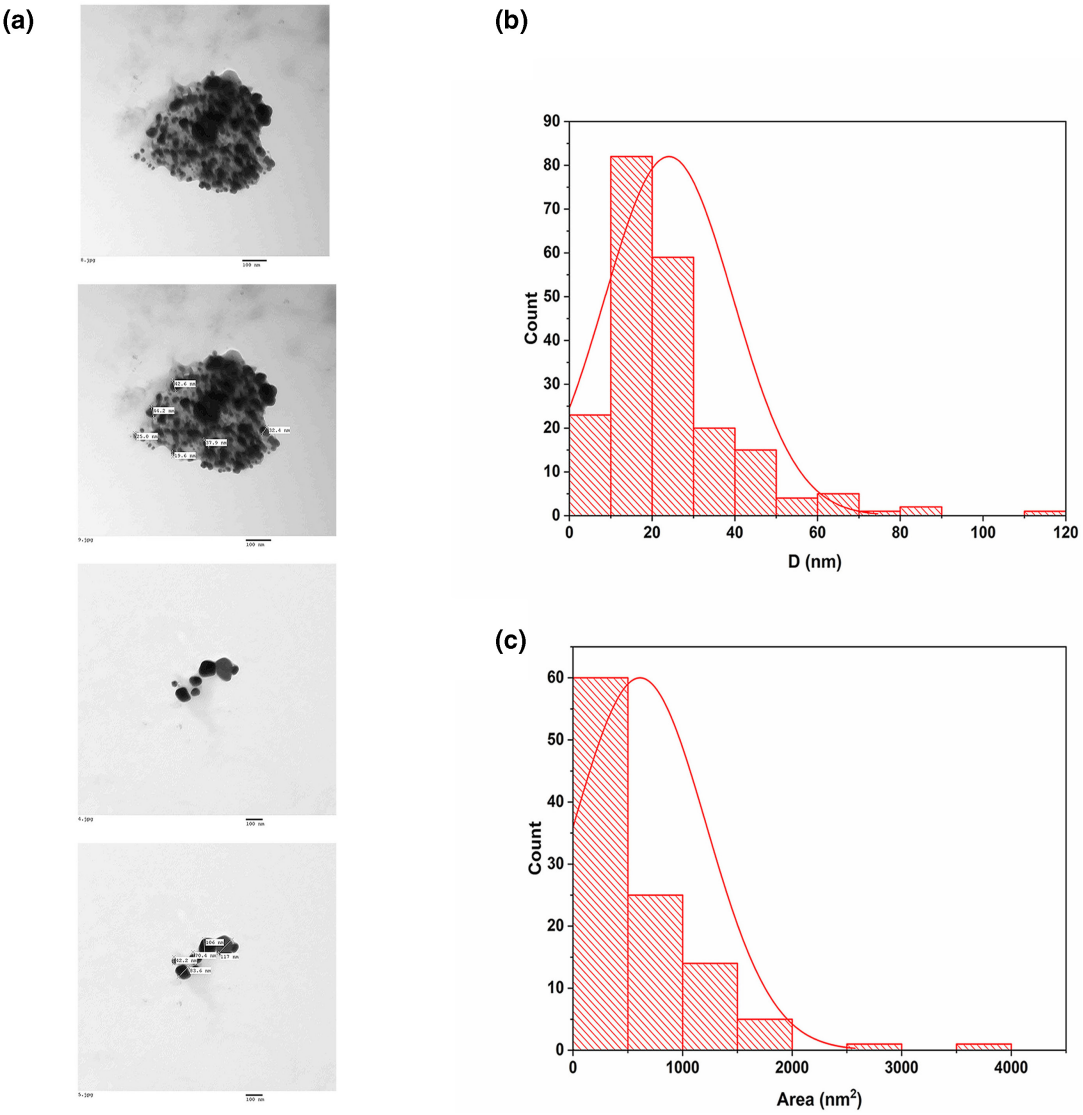
Characterization of APS‐NPs using TEM technology: (a) TEM images of APS‐NPs synthesis by using alcoholic extract of *Aloe perryi*. (b) Histogram of particle size distribution (diameter) of APS‐NPs analyzed by Image J software, (c) Histogram of area measurement of APS‐NPs analyzed by Image J software. APS‐NPs, *Aloe perryi‐*silver nanoparticles.

Nanoparticles are mainly optimized and characterized for their shape and size via TEM instrumental analysis (Anjum, 2016). Our results showed that APS‐NPs are characterized by spherical shape and nano size with a minimum size of almost 4 nm and a maximum size of 110 nm (Figure 3a), and the histogram of TEM images of diameter size and area showed an average size of almost 24 and 611 nm^2^, respectively, indicating that our developed APS‐NPs were in proper shape and size significantly (Figure 3b,c).

### 3.4 Characterization of APS‐NPs crystallinity by using XRD

**FIGURE 4:**
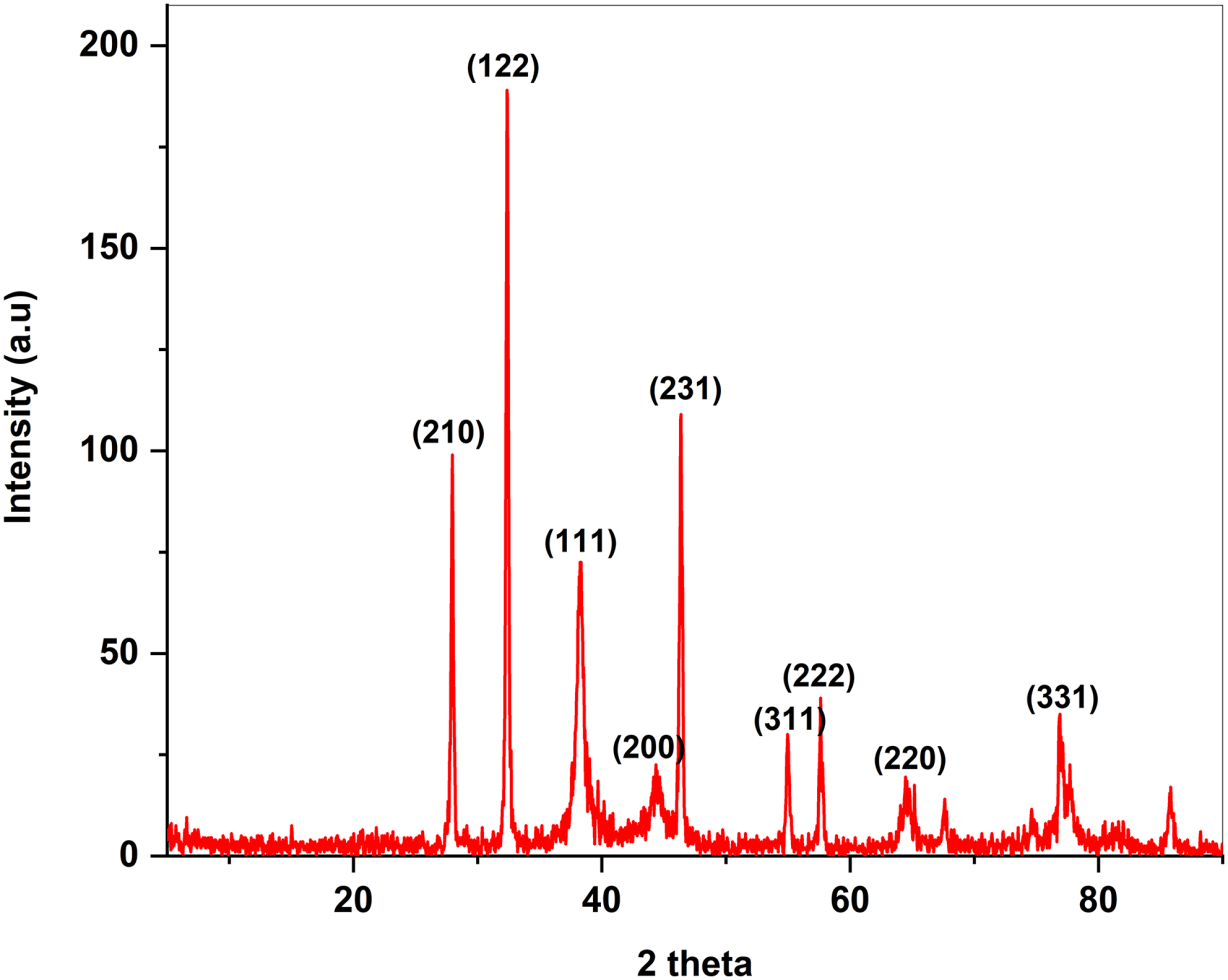
Characterization of APS‐NPs using x‐ray diffraction: x‐ray diffraction pattern of APS‐NPs synthesized by using extract of *Aloe perryi* showed APS‐NPs in cubic crystal structure and also with nano size. APS‐NPs, *Aloe perryi‐*silver nanoparticles.

In this study, the XRD pattern of the APS‐NPs results showed that prepared Ag has nine Bragg reflection peaks at 2 Theta values of 27.8°, 32.2°, 38.2°, 44.4°, 46.2°, 54.9°, 57.5°, 64.6°, and 76.6° for the growth directions (210), (122), (111), (200), (231), (311), (222), (220), and (331) (Figure 4). These nine peaks indicated and confirmed the cubic high crystallinity of our prepared APS‐NPs. The other peaks that appeared were related to the *A. perryi* extract and AgNO3 solution. The sizes of the APS‐NPs were calculated through Debye–Scherrer's equation (Table 1) and showed that the APS‐NPs average size was 21.84 nm.

**TABLE 1:** XRD values of developed APS‐NPs.

Size (nm)	FWHM(β)	Peak position(2θ)
32.89737942	0.24876	27.95775
33.3222351	0.24816	32.38557
10.00914492	0.83979	38.26862
3.388019071	2.53172	44.41677
30.11148351	0.2869	46.37309
30.57231533	0.29281	54.97835
28.2534251	0.3208	57.63633
9.104357629	1.03188	64.59328
29.9796494	0.31876	67.59898
7.846428707	1.29466	77.16107
24.76022629	0.43783	85.80208

Abbreviation: FWHM (β), full width at half maximum.

### 3.5 Characterization of APS‐NPs by using FTIR

**FIGURE 5:**
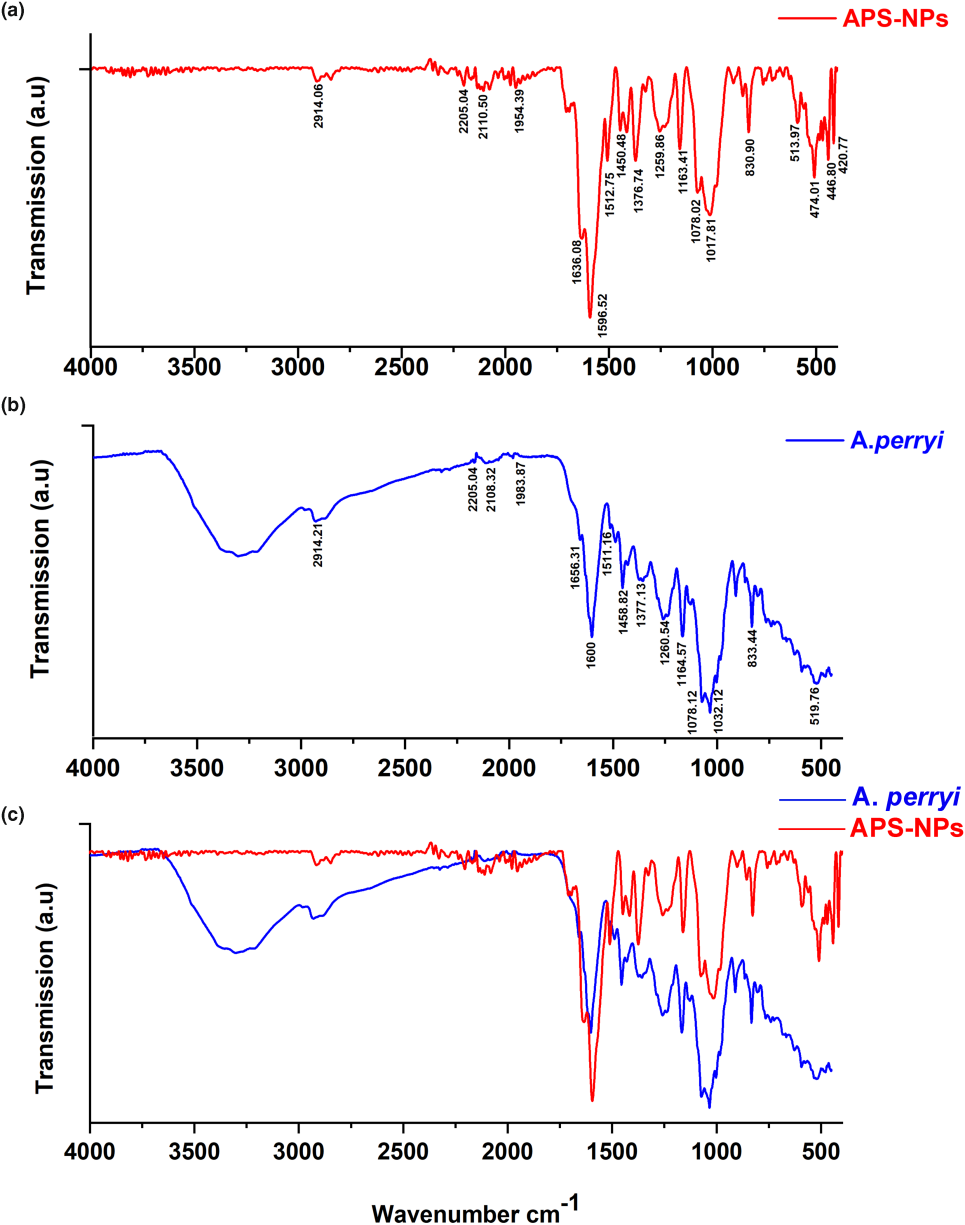
Characterization of APS‐NPs using FTIR analysis: (a) FTIR of APS‐NPs indicates functional group peaks. (b) FTIR of *Aloe perryi* extract indicates functional group peaks. (c) Merged FTIR peaks of APS‐NPs and *A. perryi* extract in this study in the range of 4000–400 cm^−1^ suggested that functional groups of APS‐NPs were related to the phytochemicals of *A. perryi* and that could play a role in reducing silver ion and the formation of APS‐NPs. APS‐NPs, *Aloe perryi‐*silver nanoparticles.

To determine and validate the presence of functional groups in the moiety component of a chemical, FTIR spectroscopy is a useful investigative technique. Scannable samples in the 4000–400 cm^−1^ range were used to identify the major functional groups in herbal extract and silver nanoparticles (Singh et al., 2013). In this study, our FTIR results indicated that the functional groups shown in Table 2 were involved in the formation of APS‐NPs (Figure 5a), and these functional groups were similar to those in *A. perryi* extract (Figure 5b). These results suggest that functional groups present in the phytochemicals of *A. perryi* extract could play a major role in reducing and capping silver ions and forming APS‐NPs (Figure 5a–c).

**TABLE 2:** FTIR values of developed APS‐NPs.

Bands (cm^−1^)	Functional group	Type of vibration	Intensity
2914.06	Alkanes CH	Stretch	Medium
2205.04	Alkynes C≡H	Stretch	Weak
2110.50	Alkynes C≡H	Stretch	Weak
1980.46	Allene C=C=C	Stretch	Medium
1954.39	Allene C=C=C	Stretch	Medium
1596.52	Cyclic alkene C=C	Stretch	Medium
1512.75	Nitro compound N‐O	Stretch	Strong
1450.48	Alkane C‐H	Bend	Medium
1418.96	Carboxylic acid O‐H	Bend	Medium
1376.74	Phenol O‐H	Bend	Medium
1259.86	Alkyl aryl ether C‐O	Stretch	Strong
1163.41	None		
1078.02	Primary alcohol C‐O	Stretch	Strong
1017.81	None		
904.65	None		
830.90	Alkene C=C	Bend	Strong
760.76	Aromatic C‐H	Bend	Strong
716.60	Alkene C=C	Bend	Strong
595.84	Halo compound C‐I	Stretch	Strong
513.97	Halo compound C‐I	Stretch	Strong
474.01	None		
446.80	None		
420.77	None		

### 3.6 Characterization of APS‐NPs charge and size by using zeta potential

**FIGURE 6:**
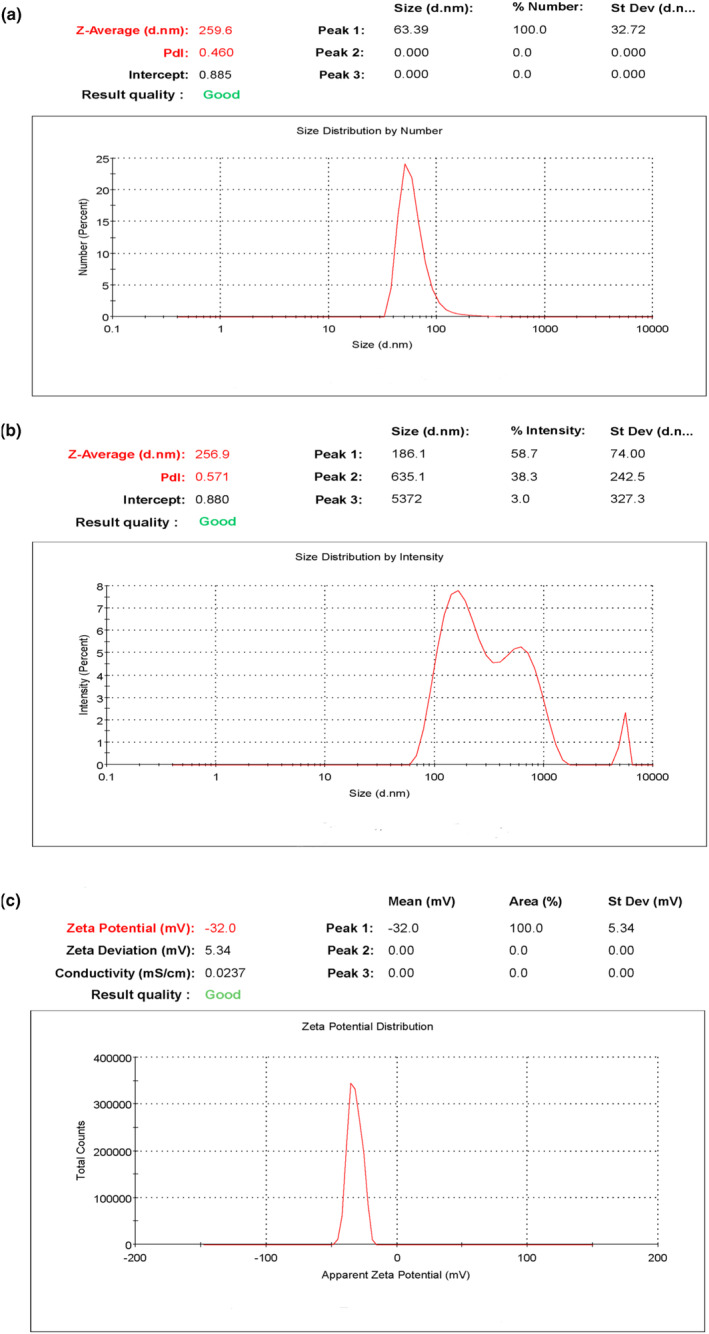
Characterization of APS‐NPs using zeta potential: (a) size distribution by number of APS‐NPs, (b) size distribution by intensity of APS‐NPs, (c) zeta potential illustrated the negative charge on the surface of developed APS‐NPs. APS‐NPs, *Aloe perryi‐*silver nanoparticles.

Zeta potential is used to assess particle surface charges and determine stability and molecular interaction (Delgado et al., 2007). In this study, our zeta potential results indicated that the majority of APS‐NPs size distribution was 63.39 nm, with a polydispersity index (PDI) value of 0.460, suggesting the proper nano size of developed APS‐NPs (Figure 6a,b). However, several studies reported that biogenic nanoparticles made using plant resources were characterized by negative zeta potentials (Nath, 2015; Raj et al., 2020). Negative low potential values might be attributed to the biomolecules encasing the nanoparticles with negatively charged functional groups, resulting in the particle's electrostatic repulsion. As a result, stable nanoparticles may be predicted (Sujitha & Kannan, 2013). Thus, our results of zeta potential analysis showed a negative value of −32 mV for the developed APS‐NPs (Figure 6c), revealing that our developed APS‐NPs were sufficiently stable.

### 3.7 APS‐NP‐induced antioxidative activity

**FIGURE 7:**
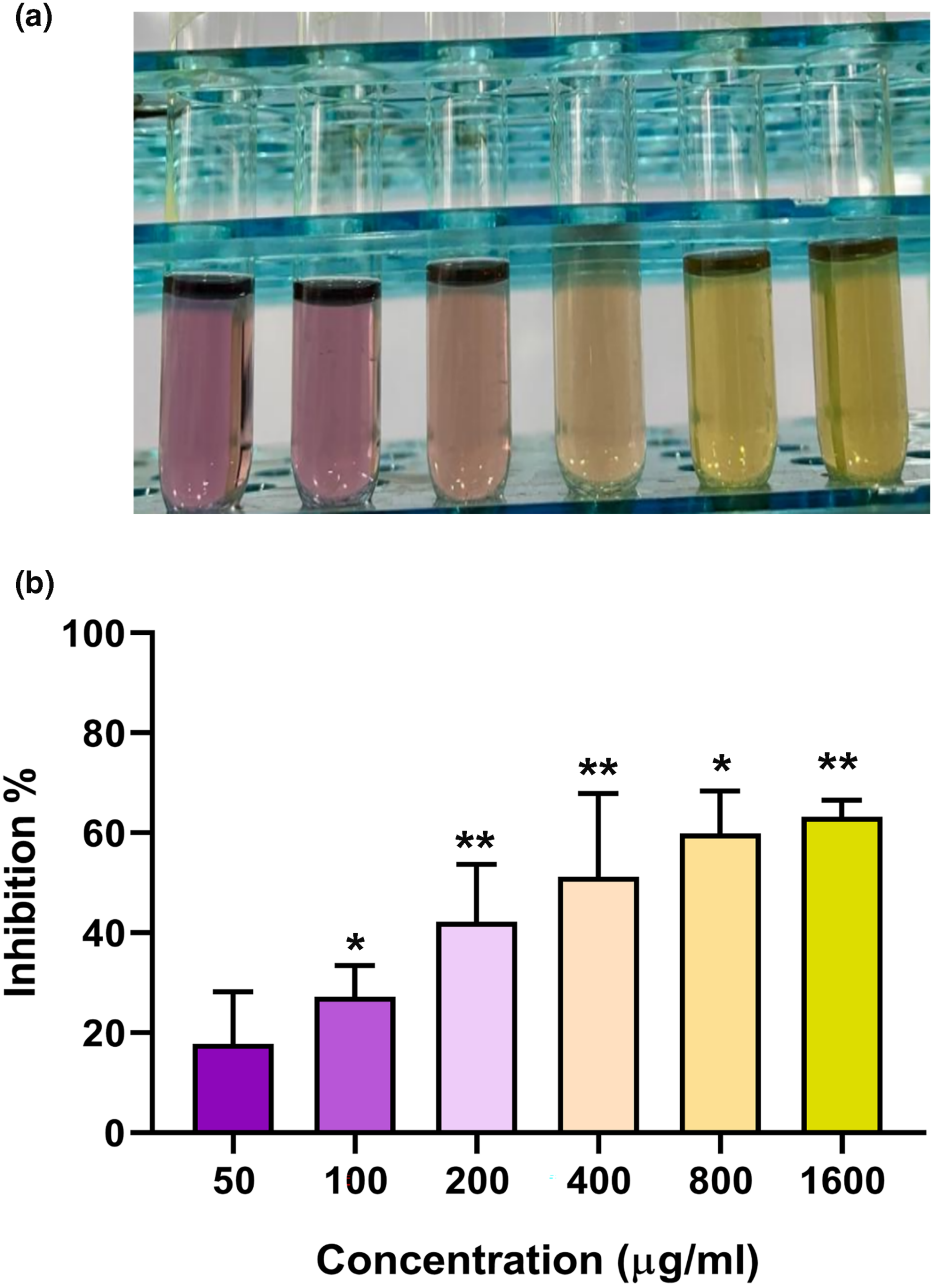
Determination of the antioxidative stress activity of APS‐NPs: (a, b) in vitro determination of APS‐NP‐induced antioxidative stress activity by inhibiting DDPH free radicles in various dilutions of APS‐NPs. The asterisks (*) and (**) illustrated a significant difference (*p* ≤ .05), (*p* ≤ .01) compared to the control based on Duncan's multiple comparison test. Data are represented as mean ± SD, *n* = 3. APS‐NPs, *Aloe perryi‐*silver nanoparticles.

To assess the antioxidative impact of the produced APS‐NPs, a DPPH‐free radical scavenging experiment was used. DPPH is a commercially available lipophilic radical that can rapidly receive an electron from an antioxidant molecule and change its color from purple to yellow at 517 nm (Kedare & Singh, 2011). The antioxidative activity of APS‐NPs was studied by using various concentrations of 50, 100, 200, 400, 800, and 1600 μg/mL. Our findings showed that the percentage of free radical scavenging activity increased in a dose‐dependent manner (Figure 7a,b), indicating that APS‐NPs had potential antioxidative stress activity and could play an essential role in disease‐induced oxidative stress.

### 3.8 APS‐NP‐induced antibacterial activity

**FIGURE 8:**
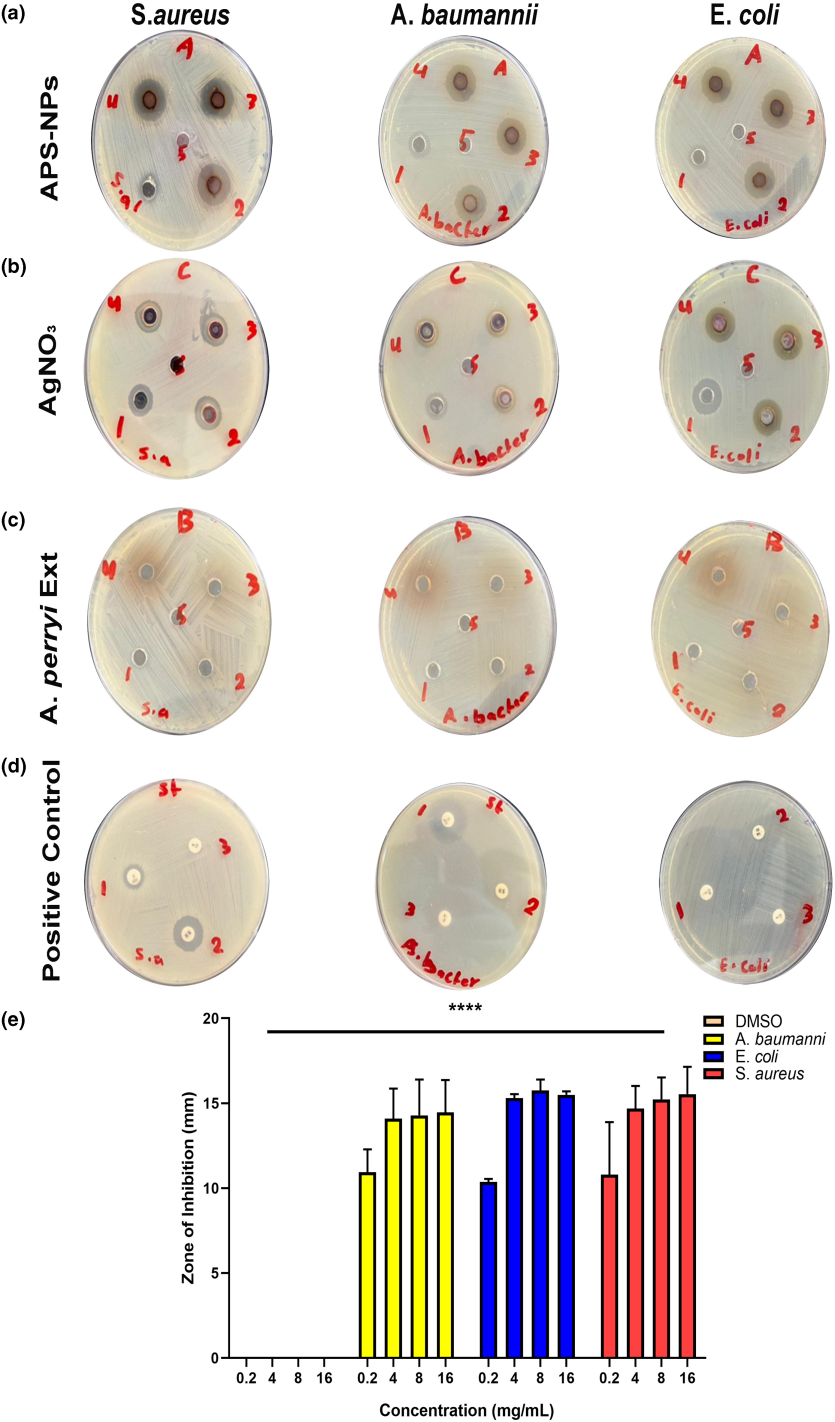
Antibacterial activity of APS‐NPs against *Acinetobacter baumannii*, *Escherichia coli*, and *Staphylococcus aureus* (a) Measurement of APS‐NP‐induced inhibition zone against *A. baumannii*, *E. coli*, and *S*. *aureus* bacteria. (b) Measurement of silver nitrate‐induced inhibition zone on bacteria against *A. baumannii*, *E. coli*, and *S. aureus*. (c) Measurement of *Aloe perryi* inhibition zone on bacteria against *A. baumannii*, *E. coli*, and *S. aureus*. All wells were filled with 50 μL of (1–4) 0.2, 4, 8, and 16 mg/mL, respectively, and (5) DMSO. (d) Measurement of positive control (standard antibiotics)‐induced inhibition zone on bacteria against *A. baumannii*, *E. coli*, and *S. aureus*, well filled with (1) Ciprofloxacin 5 μg, (2) Vancomycin 39 μg, (3) Erythromycin 15 μg. (e) quantification of measurement of bacterial growth zone inhibition by various concentrations of APS‐NPs synthesized with *A. perryi* extract after 24‐h incubation. All data were used in three independent experiments. Data are expressed as the mean ± SD (*n* = 3), ****p < .0001, versus negative control (DMSO). APS‐NPs, *Aloe perryi‐*silver nanoparticles.

In this study, our results indicated that APS‐NPs exerted antibacterial activity against various gram‐negative and gram‐positive pathogenic bacteria (*A. baumannii*, *E. coli*, and *S. aureus*) isolated from hospitalized patients (Almadeinah Medical Center, Aden, Yemen) via the well diffusion agar method. APS‐NPs in various concentrations showed a significant bacterial growth inhibition against *A. baumannii*, *E. coli*, and *S. aureus* compared to the negative control (Figure 8a; Table 3). APS‐NPs in a concentration less than 0.5 mg/mL also showed inhibition of bacterial growth against *A. baumannii* (10.95 mm), *E. coli* (10.37 mm), and *S. aureus* (10.79 mm) but not much greater than that of the concentrations of ≥4 mg/mL compared with AgNO_3_ (Figure 8b; Table 4). Both DMSO (negative control) and *A. perryi* extract (Figure 8c) did not show any inhibition of bacterial growth against all the above‐mentioned pathogenic bacteria. While against positive controls of antibiotics, our results indicated that bacterial growth (Table 5) of *S. aureus* showed intermediate sensitivity to Ciprofloxacin 5 μg (9.94 mm) and Vancomycin 30 μg (14.85 mm) and was resistant to Erythromycin 15 μg (0 mm), whereas *A. baumannii* also showed intermediate sensitivity to Ciprofloxacin 5 μg (19.25 mm), but showed resistant activity against Vancomycin 30 μg (8.72 mm) and Erythromycin 15 μg (0 mm). However, *E. coli* exerted an obvious resistance against all the antibiotics: Ciprofloxacin 5 μg (0 mm), Vancomycin 30 μg (0 mm), and Erythromycin 15 μg (0 mm), according to the CLSI values (Table 6), the standard bacterial zone of inhibition of Ciprofloxacin, Vancomycin, and Erythromycin. So, our developed APS‐NPs at the above‐mentioned concentrations inhibited bacterial growth with intermediate sensitivity of *A. baumannii*, *E. coli*, *and S. aureus*, suggesting that APS‐NPs possessed antibacterial activity against these pathogenic and infectious bacteria in Yemen.

**TABLE 3:** Measurement of inhibition zone of APS‐NPs on bacteria.

Species		Inhibition zone (mm)			*p*‐Value
Conce. (mg/mL)		*Acinetobacter baumannii*	*Escherichia coli*	*Staphylococcus aureus*	
APS‐NPs	0.2 mg/mL	10.79 mm	10.37 mm	10.95 mm	<.0001
	4 mg/mL	14.6 mm	15.3 mm	14.1 mm	<.0001
	8 mg/mL	15.2 mm	15.6 mm	14.3 mm	<.0001
	16 mg/mL	15.5 mm	15.5 mm	14.5 mm	<.0001

**TABLE 4:** Measurement of inhibition zone of AgNO_3_ on bacteria.

Species		Inhibition zone (mm)		
Conce. (mg/mL)		*Staphylococcus aureus*	*Escherichia coli*	*Acinetobacter baumannii*
AgNo3	0.2 mg/mL	12.15 mm	14.6 mm	11.52 mm
	4 mg/mL	12.52 mm	14.6 mm	11.77 mm
	8 mg/mL	12.93 mm	14.9 mm	12.02 mm
	16 mg/mL	13 mm	15.12 mm	12.39 mm

**TABLE 5:** Measurement of inhibition zone of standard antibiotics on bacteria.

Species		Inhibition zone (mm)		
Conce. μg		*Staphylococcus aureus*	*Escherichia coli*	*Acinetobacter baumannii*
Ciprofloxacin	5 μg	9.94 μg	‐	19.25 μg
Vancomycin	30 μg	14.85 μg	‐	8.72 μg
Erythromycin	15 μg	‐	‐	‐

**TABLE 6:** General standard inhibition zone of antibiotics.

Drug μg		Inhibition zone (mm)		
		Sensitive (S)	Intermediate (I)	Resistant (R)
Ciprofloxacin	5 μg	≤21 mm	16–20 mm	≤15 mm
Vancomycin	30 μg	≤17 mm	15–16 mm	≤14 mm
Erythromycin	15 μg	≤21 mm	16–20 mm	≤15 mm

### 3.9 APS‐NP‐induced cytotoxicity

**FIGURE 9:**
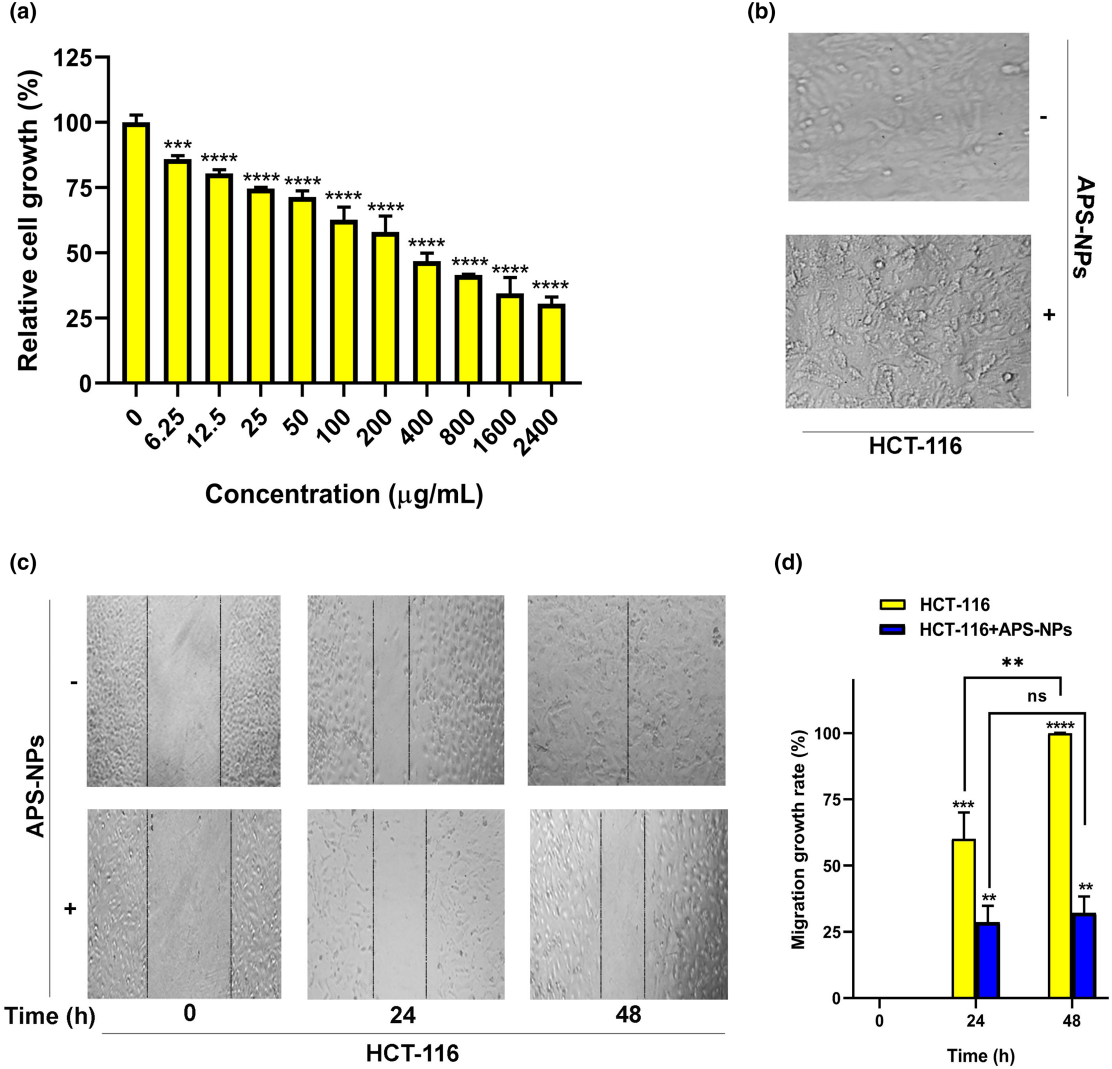
APS‐NPs induced anticancer activity against HCT‐116 cell line: (a) The effect of APS‐NPS in various dilutions (6.25, 12.5, 25, and 50, 100, 200, 400, 800, 1600, 2400 μg/mL) on HCT‐116 cells viability. (b) The effect of APS‐NPs (370 μg/mL) on HCT‐116 cell morphology. (c) The inhibitory effect of APS‐NPs (370 μg/mL) against HCT‐116 cell migration in different time points (0, 24, and 48 h) compared with control. (d) quantification analysis of migration inhibition rate of HCT‐116 cells. All data were used in three independent experiments. Data are expressed as the mean ± SD (*n* = 3), ****p < .0001, ***p < .001, and **p < .01 versus control (untreated HCT‐116). APS‐NPs, *Aloe perryi‐*silver nanoparticles.

The HCT‐116 cell line's viability was measured using the MTT test. The results indicated that APS‐NPs induced a significant gradual decrease in cell viability in treated HCT‐116 cells around 85%–33% for the concentration range of 6.25–2400 μg/mL compared with the control (100%) (Figure 9a). Furthermore, APS‐NPs not only decreased the cell viability but also APS‐NP‐induced alteration of HCT‐116 cell morphology (Figure 9b), suggesting that the developed APS‐NP‐induced cytotoxicity against colon cancer was related to the regression of cell viability and morphology.

### 3.10 APS‐NP‐induced inhibition of cell migration

A wound healing cell migration experiment was used to detect the migration ability of HCT‐116 cells. Our results indicated that APS‐NPs inhibited the HCT‐116 cell migration ability significantly in a time‐dependent manner compared with control. The rate of cell migration in control HCT‐116 cells (untreated cells) was wholly and significantly increased after 48 h (100%) compared with 0 h (0%) and 24 h (60%) (Figure 9c,d). However, treatment with APS‐NPs reduced the migration rate in HCT‐116 cells significantly in 48 h (33%) and 24 h (31%) compared (untreated cells) with control HCT‐116 cells (Figure 9c,d), indicating that inhibition of cell migration was related to APS‐NPs, suggesting that APS‐NPs possessed anticancer activity against colon cancer.

## 4 DISCUSSION

Green synthesis is considered an easy, cheap, reliable, and eco‐friendly technique that is becoming increasingly popular in the synthesis of nanoparticles (Gur et al., 2022). Once silver ions were reduced to silver nanoparticles by the plant extracts, the color of the solution would change to a deep reddish‐brown because of the surface plasmon resonance phenomenon (Balavijayalakshmi & Ramalakshmi, 2017). Our results verified our hypothesis. The mixture of *A. perryi* ethanolic extract and AgNO_3_ solution under continuous stirring and heating for 4 h resulted in a color change from orange to dark brown, suggesting the successful formation of APS‐NPs. The maximum absorption of the developed APS‐NPs was also confirmed by using ultraviolet–visible spectrophotometry, and interestingly, our APS‐NPs showed 442 nm of maximum absorption, indicating that our result was within the range of silver nanoparticles as mentioned in the literature (Ashraf et al., 2016).

Researchers have also suggested that the green synthetic silver nanoparticles, which are both tiny and spherical, have promising biological applications (Alharbi et al., 2022). Our SEM results of green synthesized APS‐NPs showed an average particle size of 16.8 nm, and the TEM images also showed an average particle size of 24 nm, revealing that our developed APS‐NPs were in nano‐regime, which could play an obvious role in therapeutic activities. XRD is one of the most commonly used techniques for determining the chemical composition and crystal structure of a material (Galatage et al., 2021). Our results of XRD indicated that the APS‐NPs developed through the reduction of silver ions by the *A. perryi* were cubic crystalline in nature, revealing the stability of that APS‐NPs structure.

Natural plants possess several phytochemical constituents that could play a role in various chemical and biological activities, which may be attributed to their functional group characteristics (Shad et al., 2014). FTIR is used to evaluate the chemical composition of silver nanoparticles' surfaces to find biomolecules for capping and stabilizing metal nanoparticles (Nisar et al., 2022). The results of FTIR of APS‐NPs indicated that functional groups detected in FTIR analysis were related to the phytochemical components of the *A. perryi* plant and suggested that they could play a main role in the reduction of silver ions into nanoparticles. Our zeta potential results were used to determine the size distribution, surface electrical charge, and stability of our developed APS‐NPs; interestingly, the zeta potentials revealed that our APS‐NPs were nano‐sized and negatively charged, indicating adequate stability.

The numerous medicinal plants used to create green‐synthesized silver nanoparticles exhibited a diverse range of pharmacological activities, including antioxidative stress, antibacterial, and anticancer properties (Husain et al., 2021; Nakkala et al., 2017). It has been reported that the antioxidative stress effect of green‐synthesized silver nanoparticles belongs to their ability to make protons donation to free radicals, and it is due to the capping of bioactive molecules of herbs on the surface of the silver nanoparticles (Kanipandian et al., 2014). Our results indicated that the APS‐NPs exerted obvious antioxidative stress activity in a dose‐dependent manner, and the DPPH radical scavenging activity was increased with increasing the APS‐NPs concentration, suggesting that the antioxidant potential of APS‐NPs encourages their application in biomedicine.

*Acinetobacter baumannii* is one of the most serious bacteria associated with a high mortality rate in hospitalized patients (Howard et al., 2012). The severity of *A. baumannii* infection is based on the site of infection and the patient's vulnerability to infection owing to the underlying disease. *Acinetobacter baumannii* induces mild‐to‐severe sickness, but it is potentially lethal (Aboshakwa et al., 2019). The occurrence of *E*. *coli* infection usually causes severe diarrhea because the occurrence of *E. coli* with multi‐drug resistance is associated with high morbidity and mortality (Dela et al., 2022). *S. aureus* causes significant community‐acquired and nosocomial illnesses in humans, from mild skin infections to septicemia (Park & Seo, 2019). Due to the severity of these bacteria, scientists keep dealing with the antibacterial activity of green‐synthesized silver nanoparticles as reported in the literature (Afzal et al., 2023; AlSalhi et al., 2019; Kumkoon et al., 2023). This study investigated the application of APS‐NPs as an antibacterial agent, it exhibited antibacterial activity against various gram‐positive and gram‐negative bacteria (*E. coli*, *A. baumannii*, *and S. aureus*). Our results indicated that APS‐NPs showed significant inhibition of growth activity of the above‐mentioned bacteria compared with positive controls, revealing their moderately sensitive activity against APS‐NPs, suggesting that APS‐NPs could be used as an antibacterial agent against serious infectious diseases.

The cytotoxic profile of green‐synthesized silver nanoparticles could be one of the most chosen anticancer agents against various human cancer cells (Wypij et al., 2021). The antitumor activity has also been investigated in this study. We have performed the MTT assay to evaluate the toxicity of APS‐NPs on HCT‐116, and the results showed that APS‐NPs induced a significant reduction in the viability of HCT 116 cells in a dose‐dependent manner, and APS‐NPs not only reduced cell viability but induced morphological alteration in the HCT 116 colon cancer cell line. Cancer cells are usually characterized by uncontrolled divisions that invade other tissues by metastasis. This phenomenon is called cell–cell migration. In the present study, we also studied the therapeutic effect of APS‐NPs on cell migration by using a scratch assay, and our results indicated that APS‐NPs exhibited significant inhibition of cell migration of treated HCT 116 cells in a time‐dependent manner compared to the control, suggesting that this inhibition of HCT‐116 cell migration by APS‐NPs could be due to the ability of APS‐NPs to interfere with the HCT‐116 cell cytoskeleton. Cell division and migration require cytoskeleton rearrangement, and interference in any vital functions notably affects cell proliferation and migration (Etienne‐Manneville, 2004). From these cytotoxicity and migration inhibition results, describing the aggressive effect of APS‐NPs against colon cancer cells might be a promising insight into the future of cancer therapy.

In conclusion, our results verified our hypothesis that *A. perryi* extract played a crucial role in synthesizing APS‐NPs as a reducing and capping agent. Our characterization studies, including ultraviolet/visible spectroscopy, SEM, TEM, XRD, and zeta potential assays, confirmed that developed APS‐NPs were stable, cubic crystalline, and nano‐sized. Furthermore, on the therapeutic level, our developed APS‐NPs exhibited pharmacological activity against oxidative stress and various infectious bacteria, as well as, APS‐NPs induced an apoptosis against human colon cancer cells, suggesting the potential use of APS‐NPs in the formulation of the pharmaceutical dosage form as antioxidative, antibacterial, and anticancer agents. The formulation of an APS‐NPs transdermal patch with *A. perryi* polymer embedded with APS‐NPs as an antioxidative and antibacterial agent against infectious disease needs further study in the future as an experimental animal study.

## AUTHOR CONTRIBUTIONS

**Marwan Almoiliqy:** Conceptualization (lead); data curation (lead); investigation (lead); project administration (lead); resources (lead); software (supporting); supervision (supporting); writing – original draft (lead); writing – review and editing (lead). **Omar Hotan:** Conceptualization (equal); methodology (equal); writing – original draft (equal). **Ali Alhaj:** Conceptualization (equal); data curation (equal); writing – original draft (equal). **Abdulghfor Al‐quhaim:** Data curation (equal); methodology (equal); writing – original draft (equal). **Khaled Alburaihi:** Methodology (equal); software (equal); writing – original draft (equal). **Yahya Ahmed:** Data curation (equal); methodology (equal). **Qassem Munasser:** Methodology (equal). **Saleh Bin Dhufer:** Methodology. **Tammam Nasran:** Formal analysis (equal). **Mohammed Gabir:** Methodology (equal). **Akram Ebrahim:** Conceptualization (equal). **Mohammed Obadi:** Software (equal). **Maryam Hadi:** Project administration (equal); resources (equal). **Hanefa Al‐baity:** Methodology (equal). **Abdulmalek Ba‐Nafea:** Software (equal). **Eskandar Qaed:** Software (equal); validation (equal); writing – review and editing (equal). **Mohamed Y. Zaky:** Validation (equal); writing – review and editing (equal). **Mohammed Okba:** Project administration (equal); writing – review and editing (equal). **Abdullah Al‐Nasi:** Writing – review and editing (equal).

## FUNDING INFORMATION

This work was supported by the University of Science and Technology Aden, Yemen, (Grant No. USTPH‐123102021).

## CONFLICT OF INTEREST STATEMENT

The authors declare that they have no conflicts of interest.

## Data Availability

Data mentioned in this work are upon request with the corresponding author.
